# A directional fibre optic ultrasound transmitter based on a reduced graphene oxide and polydimethylsiloxane composite

**DOI:** 10.1063/1.5089750

**Published:** 2019-03-21

**Authors:** R. J. Colchester, E. J. Alles, A. E. Desjardins

**Affiliations:** 1 Department of Medical Physics and Biomedical Engineering, University College London, Gower Street, London WC1E 6BT, United Kingdom; 2 Wellcome/EPSRC Centre for Interventional and Surgical Sciences, Charles Bell House, University College London, 43-45 Foley Street, London W1W 7TS, United Kingdom

## Abstract

Strongly directional ultrasound sources are desirable for many minimally invasive applications, as they enable high-quality imaging in the presence of positioning uncertainty. All-optical ultrasound is an emerging paradigm that exhibits high frequencies, large bandwidths, and a strong miniaturisation potential. Here, we report the design, modelling, and fabrication of a highly directional fibre-optic ultrasound transmitter that uses a composite of reduced graphene oxide and polydimethylsiloxane as the optical ultrasound generator. The ultrasound transmitter, which had an outer diameter of just 630 *μ*m, generated ultrasound with a pressure exceeding 0.4 MPa for axial distances up to 16 mm, at a large bandwidth of 24.3 MHz. The ultrasound beam exhibited low divergence, with a beam diameter ranging between 0.6 and 2.1 mm for distances between 0 and 16 mm. The presented directional optical ultrasound source is hence well-suited to high-resolution interventional imaging.

High resolution imaging from miniaturised devices is crucial for guidance during minimally invasive surgical procedures. Recently, all-optical ultrasound imaging has emerged as a promising modality with favourable properties. With all-optical ultrasound, ultrasound is both generated and received using light. Generation of ultrasound is achieved via the photoacoustic effect: excitation light is delivered to an optically absorbing coating where it is absorbed, leading to a temperature increase. The resulting pressure increase propagates as an ultrasound wave.^1^ Optical reception of ultrasound can be achieved using interferometric methods, where impinging back-scattered ultrasound waves result in deformations of optically resonant structures that can be accurately monitored.^2–5^ All-optical ultrasound imaging probes are readily miniaturised through the use of optical fibres and can provide broad bandwidths and high sensitivity.^6,7^ In addition, all-optical ultrasound imaging probes can be made immune to electromagnetic interference, which allows for use in electromagnetically sensitive or harsh conditions, such as inside a magnetic resonance imaging scanner or during radio-frequency ablation.^8^

Several materials have been explored for the generation of ultrasound in this context.^9^ The use of polydimethylsiloxane (PDMS) has been highlighted due to its high coefficient of thermal expansion, which ensures efficient conversion of deposited optical energy into acoustical energy and ultimately leads to the generation of high ultrasound pressures. However, PDMS is transparent over a broad wavelength range, and hence an optically absorbing component must be incorporated to enable the generation of ultrasound. Several materials have been considered, including metallic films,^10^ metallic nanoparticles,^9,11^ and carbon based materials such as carbon black,^12^ candle soot,^13^ and carbon nanotubes.^14–16^ Here, we investigate the feasibility of using reduced graphene oxide (rGO) as the optically absorbing component. Reduced graphene oxide is commercially available in a functionalised form to facilitate dissolution in organic solvents and is hence readily integrated within PDMS. In addition, rGO does not suffer from some of the health concerns associated with the elongated shape of carbon nanotubes^17,18^ and has fewer known health risks.

When deposited at the tips of miniature optical fibres, optical ultrasound generating composites have enabled both two-^6^ and three-dimensional imaging^3,9^ and real-time M-mode imaging in a clinical context.^7^ To date however, imaging has mainly been performed using weakly directional optical ultrasound sources, which either yield poor lateral resolution in M-mode imaging or involve synthetic aperture scanning and image reconstruction algorithms that require probe position measurements at an accuracy (approximately 20 *μ*m) that is not currently achievable in a minimally invasive setting.^19^

Here, we demonstrate, through simulations and experiments, an optical ultrasound source geometry that emits a highly directional ultrasound beam. In addition, we introduce a composite optical ultrasound generating material comprising reduced graphene oxide and PDMS. Given its small lateral dimensions, the resulting optical ultrasound source is well suited to integration within needles or endoscopes.

A numerical model was developed to predict the characteristics of the generated ultrasound beam. This model was based on the FOCUS simulator for MATLAB^20^ and modelled the optical ultrasound source as a circular piston for a range of diameters (200–1000 *μ*m) spanning common fibre optic core sizes (Table I). The temporal excitation of this piston was adjusted to match its frequency content to the acquired data.

**TABLE I.** Numerical study of the acoustic beam parameters for various aperture sizes. The pressure ratio is the ratio between the maximum pressure observed at the surface of the rGO/PDMS composite and at an axial distance of 16 mm.

	Aperture size (*μ*m)				
	200	400	600	800	1000
Divergence angle (°)	25.3	12.3	7.1	4.1	2.1
Pressure ratio	50.6	12.2	5.3	3.1	2.1
Beam width at 16 mm (mm)	7.7	3.9	2.6	2	1.6

Based on these simulation results, an ultrasound transmitter diameter of 600 *μ*m was found to yield a good compromise between the mechanical restrictions for minimally invasive use (size and mechanical flexibility) and the generated ultrasound beam characteristics (pressure drop-off, beam divergence, and lateral resolution). Consequently, optical fibres with core/cladding diameters of 600/630 *μ*m (FP600URT, Thorlabs, UK) were chosen. The buffer coating was stripped from the distal end and the fibre end face was polished flat. A solution of rGO functionalised with octadecylamine (805084, Sigma Aldrich, UK) and xylene was prepared by adding 500 mg of rGO to 2.5 ml of xylene. The solution was sonicated for 20 s to facilitate dispersion of the rGO in the xylene. Three prepared optical fibres were dipped into the rGO solution and removed, leaving a coating on the fibre surface. Coated optical fibres were left for 24 h in ambient conditions to dry. Subsequently, the coated fibres were dipped into a solution of PDMS (MED-1000, Polymer Systems Technology, UK) and xylene (ratio 1 g PDMS:1.8 ml xylene) and directly removed. The PDMS coated optical fibres were left to cure in ambient conditions with the distal end surface facing up to prevent the accumulation of excess PDMS at the fibre tip.

The optical fibres coated with rGO were examined visually using a stereo-microscope both before and after the application of the PDMS layer. The optical absorption of the rGO/PDMS composite coatings was measured using an integrating sphere (FOIS-1, Ocean Optics, USA), a broadband white light source (HL-2000-HP-FHSA, Ocean Optics, USA), and a spectrometer (Flame-T-VIS-NIR, Ocean Optics, USA) over a wavelength range of 345–1027 nm. An uncoated polished optical fibre was used as a reference.

The ultrasound pressure field generated by the directional optical ultrasound sources was measured using a calibrated needle hydrophone (Precision Acoustics, UK) with a diameter of 200 *μ*m and a calibration range of 1–30 MHz. Ultrasound was generated using a Q-switched Nd:YAG laser (SPOT-10–500-1064, Elforlight, UK) with a pulse width of 2 ns, a repetition rate of 100 Hz, a pulse energy of 42.3 *μ*J, and a wavelength of 1064 nm. An ultrasound field scan was performed by scanning the hydrophone over a two dimensional grid orthogonal to the longitudinal axis of the optical fibre. The grid measured 3 mm × 3 mm with an isotropic step size of 50 *μ*m and was positioned at an axial distance of 1.6 mm from the rGO/PDMS composite coating.

The measured ultrasound field scan was numerically back- and forward-propagated to determine the divergence of the ultrasound beam. For this, the angular spectrum approach^21^ was applied to propagate the ultrasound pressure field to axial distances ranging between 0 and 16 mm in steps of 1 mm.

Examination of the composite coatings with a stereo-microscope showed that the rGO coating was *ca.* 40 *μ*m in thickness (the PDMS overcoat was nearly congruent and added less than 10 *μ*m in thickness) and did not cover the entire end surface of the optical fibre. Illumination through the optical fibre showed a distinct bright ring around the edge of the coating [Fig. 1(a)]. However, the coated region was uniform and no light was visibly transmitted through the coating. For all three fibres, the optical absorption varied by less than 3% for wavelengths between 450 and 930 nm (this range was limited by the brightness of the light source), with a mean absorption between 80% and 89%. The majority of the optical transmission through the fibre could be accounted for by the uncoated ring around the edge of the optical fibre end face.

**FIG. 1.**
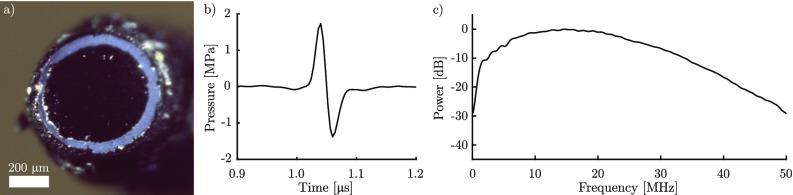
(a) Microscopic image of an optical fibre coated with an rGO/PDMS composite under white light illumination through the fibre core. (b) Ultrasound time series as measured directly in front of the rGO/PDMS optical ultrasound source at a distance of 1.6 mm. (c) The corresponding power spectrum normalised to 0 dB.

The peak ultrasound pressure in the centre of the beam at a distance of 1.6 mm from the rGO-PDMS composite coating was measured as 1.7 MPa [Fig. 1(b)]. In addition, a −6 dB ultrasound bandwidth of 24.3 MHz was found, around a peak frequency of 14.7 MHz [Fig. 1(c)]. These values are comparable to ultrasound pressures achieved with previous composite coatings.^9,13,14^

Field measurements showed that the generated ultrasound field was circularly symmetric and highly directional [Fig. 2(a)]. For axial distances ranging between 0 and 16 mm, the full-width at half-maximum (FWHM) of the ultrasound beam was found to increase from 600 *μ*m to 2.1 mm, corresponding to a divergence angle of 7° [Fig. 2(b)]. The measured ultrasound beam diameter and divergence angle matched the values predicted by simulation well. However, it was found that the simulation slightly overestimated the beam divergence.

**FIG. 2.**
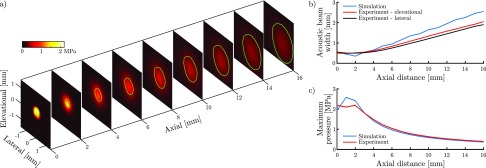
(a) The maximum pressure observed across orthogonal planes at various axial distances. The green contours represent the full-width-at-half-maximum (FWHM) of the pressure in each plane and are indicative of the acoustic beam shape. (b) The elevational (red) and lateral (black) extents of the FWHM of the acoustic beam profile as a function of axial distance. The simulated acoustic beam (blue) has rotational symmetry and hence only a single beam width is displayed. (c) The maximum pressure (in time) observed as a function of axial distance, for both simulated (blue) and measured data (red).

Due to the high degree of collimation of the optically generated ultrasound beam, the ultrasound pressure decreased only weakly with the increasing distance [Fig. 2(c)]. The peak pressure decreased from 2.2 MPa at the coating to 0.4 MPa at a distance of 16 mm and matched the simulated peak pressure closely. The discrepancies observed between measured and modelled peak pressures at axial distances below 2 mm are due to inaccuracies in the numerical back-propagation algorithm.

Here, we presented a compact optical ultrasound source that generates a highly directional ultrasound beam. The transmitter, which was based on a 600 *μ*m core diameter optical fibre, comprised a composite of reduced graphene oxide and PDMS. The high pressure, bandwidth, and degree of beam collimation found for the presented directional optical ultrasound sources suggest that these sources are well-suited to M-mode or sweep imaging, where highly collimated beams can provide high lateral imaging resolution. In addition, the slow reduction in maximum pressure with the axial distance will enable large imaging depths, and the wide bandwidth will result in high axial resolution.

Whilst the coatings fabricated here exhibited high optical absorption, inspection of the coatings showed that they did not form across the entire end face of the optical fibre, leaving a ring of uncoated fibre at the edges. For optically generated ultrasound, the acoustical bandwidth is inversely proportional to the absorbing coating thickness.^9^ While the bandwidths generated here are comparable with those reported in previous studies,^9,13,14,22^ broader bandwidths might be achieved by reducing the rGO coating thickness. This could be realised by adjusting the solvents used for the rGO coating, using similar techniques to those described in previous work using carbon nanotubes.^15^ Preliminary observations suggest that the mechanical and photo-stability of the coating are excellent, as no change in acoustical performance was observed during approximately six hours of continuous use while submerged in water.

Two-dimensional pressure field measurements were numerically back-propagated to the coating surface to yield a transduction efficiency of 0.21 MPa/(mJ/cm^2^) for the rGO-PDMS composite. This efficiency was comparable to those recently presented for carbon nanotube-based coatings^14,15^ [0.13–0.65 MPa/(mJ/cm^2^)]. However, as the latter measurements were obtained using different hydrophone dimensions and measurement distances, precise quantitative comparison is currently unfeasible.

The presented fibre-optic ultrasound transmitter based on rGO achieved a high pressure (1.7 MPa at a distance of 1.6 mm) despite a relatively low optical fluence (15 mJ/cm^2^). Assuming a similar optical damage threshold for rGO/PDMS as for carbon nanotube-based coatings,^15^ the peak pressure could be improved three-fold by increasing the pulse energy of the excitation light. Consequently, the presented directional optical ultrasound source is expected to allow for high-resolution imaging at large depths of up to a few centimeters. Additionally, the functionalised rGO used for this study was commercially available, which dramatically reduced the complexity of the fabrication process compared with previous carbon nanotube coatings that required complex functionalisation processes prior to use.^15^ Furthermore, evidence suggests that, due to its particle shape, rGO is likely to have a lower toxicity than carbon nanotubes^17^ and hence might be better suited for use in medical devices.

The optical ultrasound source presented here derived its directivity from its flat, circular aperture (600 *μ*m in diameter) that was large compared to the acoustic wavelength corresponding to the frequency content of the generated ultrasound. However, for the low frequencies (below *ca.* 6 MHz), this source is only partially directional, resulting in increased ultrasound beam divergence. In future work, the directivity of the optical ultrasound source could thus be improved by suppressing the lower frequencies, either through filtering in post-processing or by deliberately limiting the bandwidth of the excitation light using a temporally modulated light source.^23^ In addition, the divergence angle of the ultrasound beam can be further reduced by increasing the diameter of the optical fibre. However, this would decrease the lateral resolution at short axial distances because of the larger beam diameter.

The type of collimated ultrasound beam generated by the source presented here is desirable for imaging in dynamic environments where precise source positioning is challenging, such as intra-operative or interventional procedures. In such procedures, conventional omni-directional optical ultrasound sources and the associated synthetic aperture scanning and image reconstruction are impractical due to space and time constraints. Previous work utilised concave structures (with a diameter of 2 mm) to generate approximately collimated ultrasound beams.^19^ However, the planar geometry presented here achieved better collimation, higher pressures, and wider bandwidths, at a reduced overall diameter.

Similar to the previously presented concave geometry, the optical ultrasound source presented in this work uses an optical fibre with a relatively large core diameter. As such, the current device has a large minimum bend radius of 24 mm, thus making it impractical for use in some clinical scenarios. In order to overcome this limitation, a device is being designed comprising an optical fibre with a smaller core diameter and a short section of larger diameter at the distal end.

The results presented here suggest that the directional optical ultrasound source is well-suited for high-resolution M-mode imaging. Due to its flat aperture geometry, the presented optical ultrasound source lends itself to inexpensive fabrication processes with low complexity and in addition achieves better collimation at a smaller footprint than previously presented probes. Furthermore, the composite coating comprising PDMS and pre-functionalised rGO was shown to be a viable alternative to the more common carbon nanotube/PDMS composites, while requiring fewer preparation steps and likely exhibiting fewer adverse health effects. The presented directional optical ultrasound probe is hence ideally suited to biomedical and especially interventional applications.

## References

[1] P. Beard , Interface Focus 1, 602 (2011). 10.1098/rsfs.2011.0028 22866233 PMC3262268

[2] E. Zhang and P. Beard , in *Proceedings SPIE BiOS* ( International Society for Optics and Photonics, 2015), pp. 932311–932311–9.

[3] J. Guggenheim , J. Li , T. Allen , R. Colchester , S. Noimark , O. Ogunlade , I. Parkin , I. Papakonstantinou , A. Desjardins , E. Zhang , and P. Beard , Nat. Photonics 11, 714 (2017). 10.1038/s41566-017-0027-x

[4] H. Wei and S. Krishnaswamy , Opt. Lett. 42, 2655 (2017). 10.1364/OL.42.002655 28957308

[5] S. Ashkenazi , C.-Y. Chao , L. Guo , and M. O'donnell , Appl. Phys. Lett. 85, 5418 (2004). 10.1063/1.1829775

[6] R. Colchester , E. Zhang , C. Mosse , P. Beard , I. Papakonstantinou , and A. Desjardins , Biomed. Opt. Express 6, 1502 (2015). 10.1364/BOE.6.001502 25909031 PMC4399686

[7] M. Finlay , C. Mosse , R. Colchester , S. Noimark , E. Zhang , S. Ourselin , P. Beard , R. Schilling , I. Parkin , I. Papakonstantinou , and A. Desjardins , Light: Sci. Appl. 6, e17103 (2017). 10.1038/lsa.2017.103 30167220 PMC6062020

[8] E. Alles , R. Colchester , Y. Makki , S. Noimark , E. Zhang , P. Beard , M. Finlay , and A. Desjardins , in *Proceedings IEEE IUS* (2018).

[9] S. Noimark , R. Colchester , R. Poduval , E. Maneas , E. Alles , T. Zhao , E. Zhang , M. Ashworth , E. Tsolaki , and A. Chester , Adv. Funct. Mater. 28, 1704919 (2018). 10.1002/adfm.201704919

[10] T. Lee and L. Guo , Adv. Opt. Mater. 5, 1600421 (2017). 10.1002/adom.201600421

[11] X. Zou , N. Wu , Y. Tian , and X. Wang , Opt. Express 22, 18119 (2014). 10.1364/OE.22.018119 25089431

[12] T. Buma , M. Spisar , and M. O'Donnell , IEEE Trans. Ultrason., Ferroelectr. Frequency Control 50, 1161 (2003). 10.1109/TUFFC.2003.1235327 14561032

[13] W.-Y. Chang , W. Huang , J. Kim , S. Li , and X. Jiang , Appl. Phys. Lett. 107, 161903 (2015). 10.1063/1.4934587

[14] R. Colchester , C. Mosse , D. Bhachu , J. Bear , C. Carmalt , I. Parkin , B. Treeby , I. Papakonstantinou , and A. Desjardins , Appl. Phys. Lett. 104, 173502 (2014). 10.1063/1.4873678

[15] S. Noimark , R. Colchester , B. Blackburn , E. Zhang , E. Alles , S. Ourselin , P. Beard , I. Papakonstantinou , I. Parkin , and A. Desjardins , Adv. Funct. Mater. 26, 8390 (2016). 10.1002/adfm.201601337

[16] H. Baac , J. Ok , A. Maxwell , K.-T. Lee , Y.-C. Chen , A. Hart , Z. Xu , E. Yoon , and L. Guo , Sci. Rep. 2, 989 (2012). 10.1038/srep00989 23251775 PMC3524551

[17] C. Bussy , H. Ali-Boucetta , and K. Kostarelos , Acc. Chem. Res. 46, 692 (2013). 10.1021/ar300199e 23163827

[18] J. Zhang , M. Terrones , C. Park , R. Mukherjee , M. Monthioux , N. Koratkar , Y. Kim , R. Hurt , E. Frackowiak , and T. Enoki , Carbon 98, 708 (2016). 10.1016/j.carbon.2015.11.060

[19] E. Alles , S. Noimark , E. Zhang , P. Beard , and A. Desjardins , Biomed. Opt. Express 7, 3696 (2016). 10.1364/BOE.7.003696 27699130 PMC5030042

[20] R. McGough , “ FOCUS: Fast object-oriented C++ ultrasound simulator,” see http://www.egr.msu.edu/fultras-web/.

[21] X. Zeng and R. McGough , J. Acoust. Soc. Am. 125, 2967 (2009). 10.1121/1.3097499 19425640 PMC2806438

[22] T. Lee , H. Baac , Q. Li , and L. Guo , Adv. Opt. Mater. 6, 1800491 (2018). 10.1002/adom.201800491

[23] E. Alles , R. Colchester , and A. Desjardins , IEEE Trans. Ultrason., Ferroelectr. Frequency Control 63, 83 (2016). 10.1109/TUFFC.2015.2497465 26552084

